# Assessment of inverse publication bias in safety outcomes: an empirical analysis

**DOI:** 10.1186/s12916-024-03707-2

**Published:** 2024-10-25

**Authors:** Xing Xing, Jianan Zhu, Linyu Shi, Chang Xu, Lifeng Lin

**Affiliations:** 1 Department of Biostatistics, Johns Hopkins Bloomberg School of Public Health, Baltimore, MD USA; 2 Department of Biostatistics, School of Global Public Health, New York University, New York, NY USA; 3 AbbVie Inc, North Chicago, IL USA; 4 Proof of Concept Center, Eastern Hepatobiliary Surgery Hospital, Third Affiliated Hospital, Second Military Medical University, Naval Medical University, Shanghai, China; 5 Department of Epidemiology and Biostatistics, University of Arizona, Tucson, AZ USA

**Keywords:** Adverse event, Funnel plot, Inverse publication bias, Publication bias, Systematic review

## Abstract

**Background:**

The aims of this study were to assess the presence of inverse publication bias (IPB) in adverse events, evaluate the performance of visual examination, and explore the impact of considering effect direction in statistical tests for such assessments.

**Methods:**

We conducted a cross-sectional study using the SMART Safety, the largest dataset for evidence synthesis of adverse events. The visual assessment was performed using contour-enhanced funnel plots, trim-and-fill funnel plots, and sample-size-based funnel plots. Two authors conducted visual assessments of these plots independently, and their agreements were quantified by the kappa statistics. Additionally, IPB was quantitatively assessed using both the one- and two-sided Egger’s and Peters’ tests.

**Results:**

In the SMART Safety dataset, we identified 277 main meta-analyses of safety outcomes with at least 10 individual estimates after dropping missing data. We found that about 13.7–16.2% of meta-analyses exhibited IPB according to the one-sided test results. The kappa statistics for the visual assessments roughly ranged from 0.3 to 0.5, indicating fair to moderate agreement. Using the one-sided Egger’s test, 57 out of 72 (79.2%) meta-analyses that initially showed significant IPB in the two-sided test changed to non-significant, while the remaining 15 (20.8%) meta-analyses changed from non-significant to significant.

**Conclusions:**

Our findings provide supporting evidence of IPB in the SMART Safety dataset of adverse events. They also suggest the importance of researchers carefully accounting for the direction of statistical tests for IPB, as well as the challenges of assessing IPB using statistical methods, especially considering that the number of studies is typically small. Qualitative assessments may be a necessary supplement to gain a more comprehensive understanding of IPB.

**Supplementary Information:**

The online version contains supplementary material available at 10.1186/s12916-024-03707-2.

## Background

Randomized clinical trials (RCTs) offer a powerful tool to evaluate the benefits and risks of medical interventions; however, they are often hampered by financial burdens and complex implementation [1]. Meta-analyses are widely used to summarize the results from different RCTs and provide an overall effect estimate, underpinning guidelines in evidence-based medicine [2]. The quality of meta-analytical results depends highly on the risk of bias of the studies included in systematic reviews. The risk of bias occurs at both the within- and between-study levels. Publication bias (PB) is a type of between-study bias, and it generally refers to the preference for publishing studies based on the nature and direction of their findings, for example, those with significant results or large effect sizes [3, 4]. PB in the medical field challenges the validity of evidence synthesis, may lead to incorrect clinical decision-making, and could thus influence evidence-driven medical practice and result in the wastage of medical resources.

While efficacy outcomes of drugs are usually the focus of evidence research, adverse events also play a crucial role in determining the overall safety profile and acceptability of a medication in clinical practice. Assessing safety outcomes is an essential component of systematic reviews and meta-analyses, yet it is often inadequately reported [5–7]. Recently, Ioannidis discussed the phenomenon of inverse publication bias (IPB) [8], where studies without significant results, i.e., no harmful effects on patients, are more likely to be published. Detecting PB in the assessment of safety outcomes is crucial for ensuring a safe environment in patient care.

The data of adverse events may suffer from low event rates, sometimes leading to many zero events and limited sample size, challenging the detection of IPB [9–11]. A previous study in 2015 assessed PB in meta-analyses from the *Cochrane Database of Systematic Reviews* [12]; it found that results showing no evidence of adverse effects were, on average, 78% (95% credible interval: 51 to 113%) more likely to be included than results showing adverse effects in meta-analyses of safety. Another recent study also empirically assessed PB but only included a small number of trials and drugs with a focus on efficacy [13]. To the best of our knowledge, the existing empirical studies on PB did not particularly focus on the assessment of IPB of adverse events, using methods that should be tailored to adverse events. For example, while the usual PB in efficacy outcomes tends to bias away from the null effect, IPB in adverse events favors results closer to the null value.

Motivated by a large empirical dataset of meta-analyses of adverse events established by our team recently, this cross-sectional study aims to (1) investigate the presence of IPB in adverse events; (2) evaluate whether researchers can consistently identify IPB through visual inspection of funnel plots; and (3) explore the impact of considering versus disregarding effect direction in assessing IPB.

## Methods

### Date resources

The SMART Safety is an empirical dataset for evidence synthesis of adverse events [14]. It includes a systematic evaluation of RCTs focused on healthcare interventions where adverse events were the only outcome [14]; all meta-analyses in the dataset consist of trials examining safety outcomes. Specifically, the dataset contains 151 systematic reviews with 629 meta-analyses, covering more than 2300 RCTs and 362 harm outcomes, with 10,069 rows and 45 columns of trial-level information. Systematic reviews of drug safety for binary outcomes published between 1 January 2015 and 1 January 2020 were searched for and collected based on RCTs with at least one paired meta-analysis of at least five trials reporting 2 × 2 table data for each trial, with no restriction on region, treatment regimen, or type of side effect [15, 16]. Although the dataset is limited to a specific time range, this range was selected considering the timeline of a previous study [12]. Our database has undergone several rounds of proofreading, establishing itself as the largest database for systematic reviews of drug safety.

Data extraction was conducted separately for two levels of analysis. For trial-level data used in this study, information such as 2 × 2 table data was collected. Any potential errors in data extraction for the meta-analysis were resolved by cross-referencing the original trial sources, including full-text articles, their supplements, ClinicalTrials.gov, or pharmaceutical company websites. At the systematic review level, relevant information (e.g., region) was collected.

Additionally, we collected information on how IPB was assessed in each selected systematic review, with two authors (XX and JZ) independently conducting this process. The term “inverse publication bias” is not widely used in current systematic reviews, and many reviews tend to treat IPB of safety outcomes similarly to classic PB. In light of this, to determine whether IPB was assessed in the original articles, we searched for the terms “publication bias” or “small study effect” within the main text and supplementary materials. For systematic reviews that did assess IPB, we also extracted details regarding the significance levels of IPB definitions and the methods used for assessment.

To address the issue of missing data in systematic reviews, trials with missing 2 × 2 table data in either the treatment arm or control arm were excluded from our analysis. To be included in the current analysis, eligible systematic reviews from the SMART Safety dataset were required to have at least one meta-analysis with a minimum of 10 trials, ensuring sufficient statistical power [17].

### Visual assessment of IPB

We conducted the visual assessment using contour-enhanced funnel plots and trim-and-fill funnel plots for IPB [18, 19]. The trim-and-fill funnel plot incorporates both published studies and potentially missing studies, which are imputed using the trim-and-fill method. The asymmetry was assessed by examining imputed studies. More asymmetric funnel plots typically indicate more severe bias, but the asymmetry can be caused by various factors, such as heterogeneity due to subgroup effects or chance asymmetry when the number of studies is small. Contour-enhanced funnel plots are particularly useful for distinguishing PB, as well as IPB, from potential confounders [20]. They use contours to depict the significance levels of studies, which are crucial for ascertaining IPB. For instance, if a funnel plot’s asymmetry is caused by missing studies close to null effects, then such asymmetry is unlikely an indicator of IPB. Given the limited sample sizes often seen in adverse events, we also employed sample-size-based funnel plots as a sensitivity analysis for the visual tests [21].

We chose the odds ratio (OR) as the primary effect measure for adverse events based on recommendations from previous studies [22, 23]. The OR is a popular effect measure for meta-analysis due to its favorable statistical properties; various methods for assessing PB of binary outcomes are based on ORs [24–26]. We used the “meta” package (version 7.0–0) in R (version 4.3.0) to generate the contour-enhanced and trim-and-fill funnel plots. Additionally, we employed the R package “altmeta” (version 4.1) for sample-size-based funnel plots. Sample R code used for funnel plot generation is accessible on the Open Science Framework (https://osf.io/kgez5/).

Despite the wide use of funnel plots, intuitively identifying PB through visual inspection can be challenging for researchers, and their conclusions may be subjective [27]. To assess the reliability of identifying IPB through visual inspection of funnel plots, we evaluated the agreement between two independent assessors (XX and JZ). We categorized the level of IPB into four levels: “no IPB,” “mild IPB,” “moderate IPB,” and “severe IPB.” The assessors were asked to classify each meta-analysis in our dataset into one of these four levels based on the aforementioned types of funnel plots. The agreement rate between the two independent assessors was measured by the kappa statistic.

### Quantitative assessment of IPB

The quantitative assessment was conducted through Egger’s test and Peters’ test [24, 28]. Egger’s test is a straightforward and commonly used method for asymmetry detection for generic effect measures. It uses a weighted regression for effect sizes against their standard errors. In contrast, Peters’ test is developed for binary outcomes, and it avoids the mathematical association between the log OR and its standard error. In situations involving rare events or small sample sizes, Egger’s test may lead to inflated false positive rates in claiming the existence of PB, whereas Peters’ test can effectively control these rates. We assessed the agreement between the two tests.

Furthermore, as indicated in previous research, the effect direction is a critical factor in PB assessments [29, 30]. In adverse outcome research, studies with smaller effect sizes are more likely to be published, indicating that missing studies are more likely to be found on the right side of the funnel plot. Consequently, hypothesis testing should be one-sided, as opposed to the traditional two-sided *P* value approach for PB testing. To emphasize the importance of the direction in assessing IPB in our dataset of adverse events, we conducted both one- and two-sided tests to determine the number of IPB conclusions that shifted from significant to non-significant or the reverse. We used kappa statistics to evaluate the pairwise agreement rates among the foregoing quantitative methods.

We set the statistical significance threshold for IPB at 0.1 [31]. Egger’s and Peters’ tests were performed using the aforementioned R packages “meta” and “altmeta.” Excel 2021 (Microsoft, Washington) and the R package “venn” (version 1.12) were used to visualize the quantitative results.

### Handling of zero event counts

Zero event counts are a common occurrence for rare adverse events, yet they present challenges in meta-analysis. In our analysis, we addressed zero event counts for each comparison within each study in accordance with the Cochrane Handbook guidelines. For studies with only a single arm containing no events, a continuity correction of 0.5 was added to all four cells in the associated 2 × 2 table. Regarding studies with zero events in both arms, there is an ongoing debate within the research community about whether to include or exclude them from meta-analyses. While sophisticated methods, such as generalized mixed effects models and Bayesian hierarchical models, exist to incorporate these studies, current methods for assessing PB or IPB are not well-equipped to handle them effectively. Consequently, we excluded these studies from our assessments of IPB [32, 33].

## Results

### Characteristics of the SMART Safety dataset

**Fig. 1:**
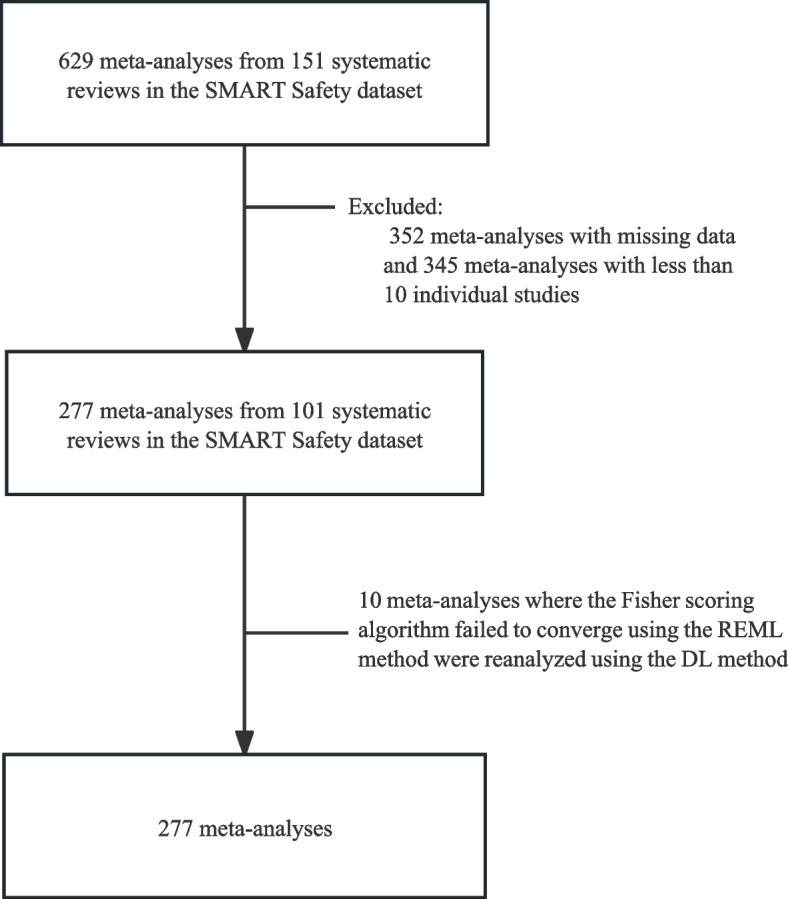
Process of identifying and analyzing the eligible data from the SMART Safety dataset

From the SMART Safety dataset, we identified 277 meta-analyses of safety outcomes, each consisting of at least 10 trials after excluding records with missing data; these meta-analyses originated from 101 systematic reviews. However, 10 meta-analyses could not be calculated due to the non-convergence of the Fisher scoring algorithm when using the restricted maximum-likelihood (REML) method, which was our primary and preferred method for estimating between-study variance [34]. To address this computational issue, we switched to the DerSimonian–Laird (DL) method for variance estimation. The process of identifying and analyzing these datasets is illustrated in Fig. 1. The median number of studies included in these meta-analyses was 13.

We evaluated the approaches to IPB detection in original articles. Of the 101 systematic reviews, 11 (10.9%) did not mention detecting IPB, and 38 (37.6%) relied solely on funnel plots for assessing IPB. Among 51 articles employing quantitative assessments, Egger’s test was the most commonly used method (45; 88.2%). Begg’s test and Harbord’s test were utilized in 30 (58.8%) and 4 (7.8%) articles, respectively. Only 4 (7.8%) systematic reviews used the trim-and-fill method, while none employed Peters’ test for IPB detection. Regarding the significance threshold, most systematic reviews employed a two-tailed *P* value of less than 0.05. Only one article specified the significance level for IPB as a *P* value < 0.1. The details of IPB detection from the original articles are presented in Additional file 1: Table S1.

Overall, all approaches suggest the presence of some IPB in the SMART dataset. Visual assessments identified between 19 (6.9%) and 40 (14.4%) meta-analyses with IPB, based on visual evaluations by two assessors. The one-sided Egger’s test and Peters’ test yielded similar proportions of IPB, with 38 (13.7%) and 45 (16.2%) meta-analyses, respectively (Additional file 2: Fig. S1). In contrast, the two-sided Egger’s test and Peters’ test indicated that approximately 80 (28.9%) and 46 (16.6%) meta-analyses, respectively, exhibited IPB. Detailed results of the visual and quantitative assessments of IPB are as follows.

### Visual assessment of IPB

The contour-enhanced funnel plots, trim-and-fill funnel plots, and sample-size-based funnel plots were made available on the Open Science Framework (https://osf.io/kgez5). Additional file 1: Table S2 shows the results of the visual assessment by two authors. For the level of “severe IPB,” assessor 1 defined 40 (14.4%) meta-analyses, and assessor 2 identified 19 (6.9%) meta-analyses, with only 8 meta-analyses showing agreement between them. For the level of “moderate IPB,” the two assessors provided similar counts of meta-analyses: 35 (12.6%) and 29 (10.5%), respectively, with 11 meta-analyses showing concordant results. For the level of “mild IPB,” there was a significant discrepancy in the number of meta-analyses classified by each assessor: assessor 2 categorized 136 (49.1%) meta-analyses in this group, while assessor 1 defined 57 (20.6%) meta-analyses. A total of 42 meta-analyses were in agreement in this category. Regarding the group of “no PB,” a high level of agreement was observed between the two assessors, with 81 meta-analyses showing concordant results. The kappa statistic for this assessment was 0.294, indicating a fair overall agreement.

To further evaluate the robustness of our results, we implemented binary classifications, combining the no and mild IPB levels into a single group to indicate the absence of IPB and the moderate and severe IPB levels into another group to represent the presence of IPB, resulting in a kappa statistic of 0.474. The merging of IPB categories resulted in a change from a fair to moderate agreement. The notable discrepancies between the visual assessments of the two assessors suggested the difficulty of using visual assessments to evaluate IPB.

### Quantitative assessment of IPB

We conducted four quantitative methods to assess potential IPB. The disagreement observed in the visual assessment persisted across the different quantitative methods. Regarding the agreement between the two one-sided tests, 43 (15.5%) meta-analyses showed differing IPB results. However, the two-sided tests exhibited an even higher rate of inconsistency, with 72 meta-analyses (26.0%) showing different results. The kappa statistic was 0.391 for the one-sided tests and 0.276 for the two-sided tests.

**Fig. 2:**
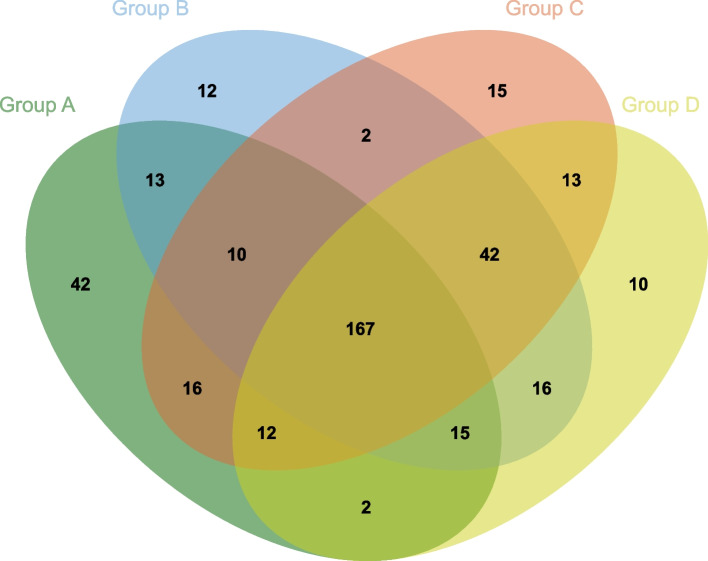
Venn diagram depicting four types of quantitative assessments. Group A: two-sided Egger’s test results; group B: two-sided Peters’ test results; group C: one-sided Egger’s test results; group D: one-sided Peters’ test results. The numbers within the overlapping areas represent the agreement between the results from the corresponding groups (tests). The total number of meta-analyses assessed for IPB is 277, which is reflected in the sum of the numbers in each group

To further explore the agreement and disagreement across the four groups, we used Venn diagrams to illustrate the associations. Additional file 2: Fig. S2 shows the agreement of non-significant results across the four groups; 182 meta-analyses showed consistent results between the one- and two-sided Egger’s tests, while 213 meta-analyses showed agreement between the one- and two-sided Peters’ tests. Additional file 2: Fig. S3 illustrates the agreement of significant results across the four groups; 23 meta-analyses had consistent results between the one- and two-sided Egger’s tests, and 27 meta-analyses showed agreement between the one- and two-sided Peters’ tests. Without classifying significant and non-significant results, the Venn diagram in Fig. 2 shows the overall agreement among the four groups: 205 (74.0%) meta-analyses showed consistent results between the one- and two-sided Egger’s tests, while 240 (86.6%) meta-analyses exhibited consistency in Peters’ tests.

The detailed results of one-sided and two-sided Egger’s test and Peters’ test are given in Additional file 1: Table S3. The kappa statistic for the one-sided and two-sided Egger’s tests is 0.250, while for the one-sided and two-sided Peters’ tests, it is 0.514. When comparing the agreement of one- and two-sided tests, 72 (26.0%) meta-analyses changed their IPB detection results after applying the two-sided Egger’s test, while 37 (13.4%) meta-analyses changed their significance in the two-sided Peters’ test.

Among the 72 meta-analyses showing differing IPB results between the one- and two-sided Egger’s tests, 15 (20.8%) changed from non-significant to significant after applying the one-sided test, while 57 (79.2%) shifted from significant to non-significant. In the case of Peters’ test, among the 37 meta-analyses with altered results, 18 (48.6%) shifted from non-significant to significant after using the one-sided test, and 19 (51.4%) shifted from significant to non-significant (Additional file 2: Fig. S4). Regarding disagreements between one-sided tests, 25 of 43 meta-analyses shifted from non-significant to significant after switching from Egger’s test to Peters’ test, showing a more balanced rate than the two-sided test (53 of 72 meta-analyses changed from non-significant to significant). Overall, all quantitative assessment methods exhibited a high level of inconsistency, suggesting the challenges of assessing IPB using these methods.

## Discussion

This study is the first to identify IPB in the largest dataset for adverse event evidence synthesis, revealing that 13.7–16.2% of meta-analyses showed IPB based on one-sided tests. Egger’s two-sided test may increase false negatives in IPB detection, while Peters’ test offered a more balanced rate. One-sided tests showed stronger alignment with both Egger’s and Peters’ tests. Of note, 10.9% of articles did not address IPB detection, and most reviews relied on funnel plots without accounting for the direction of bias. Overall, all methods of assessing IPB demonstrated a high inconsistency rate and were greatly constrained by the challenge of having a small number of studies in most meta-analyses.

IPB was present in several meta-analyses from the SMART Safety dataset. According to our quantitative assessment, the presence of IPB was not particularly high. The actual IPB status depends on many factors, such as heterogeneity. The median number of studies was 13 in our dataset. A relatively small number of studies may limit the power of statistical tests. Previous research showed that the *P* value tests could underestimate the presence of PB [31]. Therefore, the true proportion of IPB in adverse events is expected to be higher than our observed results.

For the assessment of IPB, no single method maintains optimal performance across all settings [35, 36]; all statistical methods require specific assumptions regarding the nature of the published and unpublished studies. Readers interested in a comprehensive review of the available methods for assessing IPB may refer to Xing et al. [37]. For the binary outcomes, especially for rare events, the effect measure estimates within individual studies could be mathematically related to their standard errors. Many popular methods use the association between effect estimates and the corresponding standard errors as an indicator of PB, which could cause an intrinsic association to be stronger when an event rate becomes lower and thus lead to severe inflation of type I error rates for bias detection [24, 38, 39]. Moreover, we did not use selection models to assess IPB in this study. For IPB of adverse events, the use of selection models might be challenging because adverse events are often not primary endpoints and do not directly influence the decision to publish a study. Additionally, when modeling IPB, the weight function for publication status should differ from the selection models for the classic PB [40, 41], while no consensus exists for selecting such a weight function for adverse events.

The direction of IPB is very important in IPB detection. In our study, we find more studies shifted from non-significant to significant after using the one-sided test in Egger’s test. The significant difference between one-sided and two-sided tests illustrates the need for further research. Egger’s test was the most used method (88.2%) according to our empirical study, and a two-sided test was almost universally used in each article. In practice, there is usually a specific direction for favorable results being published when comparing a new treatment vs. control [30]. When dealing with adverse events, this bias direction is expected to lean towards results closely aligned with null values or those revealing minimal adverse events within the experimental group. It is crucial for researchers to carefully consider the direction of the test and ensure adequate reporting in their review articles.

Our results indicated that it is challenging to use the visual inspection based on funnel plots for systematic reviewers to identify IPB in practice. Moreover, the agreement between the studies with mild IPB or no IPB was higher than the results for severe or moderate IPB. The poor performance of visual inspection may be due to the small number of studies. It is typically infeasible to distinguish chance from real asymmetry in meta-analyses with < 10 studies [17]. Our results in the visual assessment of IPB align with previous research [27], highlighting the high risk of error when interpreting funnel plots visually.

Our study has several strengths. First, it is a large empirical study on IPB in adverse events; it is a natural follow-up to the previous empirical study in 2015 [12]. The SMART Safety dataset is the largest dataset for evidence synthesis of adverse events, allowing us to estimate the size of IPB accurately. Second, we conducted widely used methods to detect IPB, enhancing the reproducibility and interpretability of the results. Our study provided an easy-to-follow template for future evidence-based researchers on IPB detection in adverse events. Third, our study explored the impact of considering versus disregarding effect direction in assessing IPB. Evidence from both simulated and real-world data has justified that the bias direction should be accounted for in the statistical methods for PB and IPB [29]. Fourth, we illustrated the high risk of error when interpreting funnel plots through visual examination, reminding evidence-based researchers to be aware of the limitations of the funnel plot.

The study also has several limitations. First, the true extent of IPB in this dataset is unknown, and our evidence-based findings offer supportive evidence rather than definitive conclusions for exploring IPB. Our investigation was limited to published data, as retrieving unpublished data is typically infeasible [42]. Second, many factors may contribute to the presence of IPB [43]. Owing to the large number of meta-analyses, our study does not allow us to determine the extent to which each of these factors was responsible for the observed bias. Third, due to methodological reasons, we only included meta-analyses with at least 10 studies. The size of IPB may differ in smaller meta-analyses of adverse events. Fourth, we considered only the OR as the effect measure in our study; however, the various choices of effect measures have different patterns of between-study heterogeneity, which could affect the assessment of IPB [44, 45]. Fifth, one-sided tests might result in more false negatives in detecting IPB among safety outcomes compared to two-sided tests, depending on the mechanisms causing the suppression of safety outcomes. Further exploration of the performance of one-sided tests in assessing IPB is crucial for future research.

Last but not least, double-zero event studies were removed from our analyses. Recent studies have indicated that these studies may contain important information [46]. However, most existing methods for assessing PB are based on the conventional meta-analysis framework, which requires the removal of double-zero-event studies. Because double-zero-event studies show null effects, removing these studies might lead to underestimated IPB. Bayesian approaches offer a valuable tool for incorporating such studies into meta-analyses, particularly in cases of rare adverse events [47–49]. While these approaches have primarily focused on synthesizing evidence from individual studies, little attention has been given to developing Bayesian methods for addressing PB or IPB [50]. More methodological research within the Bayesian framework is needed to properly assess and adjust for IPB in safety outcomes.

## Conclusions

In this large-scale cross-sectional study on adverse events, we found evidence of IPB in the SMART Safety data. Our findings also implied the challenges of assessing IPB using statistical methods, including both visual and quantitative assessments, especially when the number of studies is small. The true level of IPB may be even higher due to the low statistical power of the available methods.

## Supplementary Information

Additional file 1: Table S1. Summary of methods for detecting IPB in published systematic reviews. Table S2. The visual assessment results of two assessors. Table S3. The quantitative assessment conducted by the one-sided and two-sided Peters’ test and Egger’s test.

Additional file 2: Fig. S1. Proportion of significant or non-significant results in different groups. Fig. S2. Venn diagram depicting four types of quantitative assessments of non-significant results. Fig. S3. Venn diagram depicting four types of quantitative assessments of significant results. Fig. S4. Proportion of changes in significance across different groups.

## Data Availability

The SMART Safety is publicly available at https://osf.io/g3mdu. The full results of our assessment of inverse publication bias in the SMART Safety are available at https://osf.io/kgez5.

## Abbreviations

RCT: Randomized clinical trial
PB: Publication bias
IPB: Inverse publication bias
OR: Odds ratio
