# Supplementary material for: Assessment of inverse publication bias in safety outcomes: an empirical analysis
Source: BMC Med. 2024 Oct 25;22:494. doi: 10.1186/s12916-024-03707-2 (PMC11515227; doi:10.1186/s12916-024-03707-2)
Supplement: Supplementary file 2 — Additional file 2: Fig. S1. Proportion of significant or non-significant results in different groups. Fig. S2. Venn diagram depicting four types of quantitative assessments of non-significant results. Fig. S3. Venn diagram depicting four types of quantitative assessments of significant results. Fig. S4. Proportion of changes in significance across different groups. [file 12916_2024_3707_MOESM2_ESM.pdf]

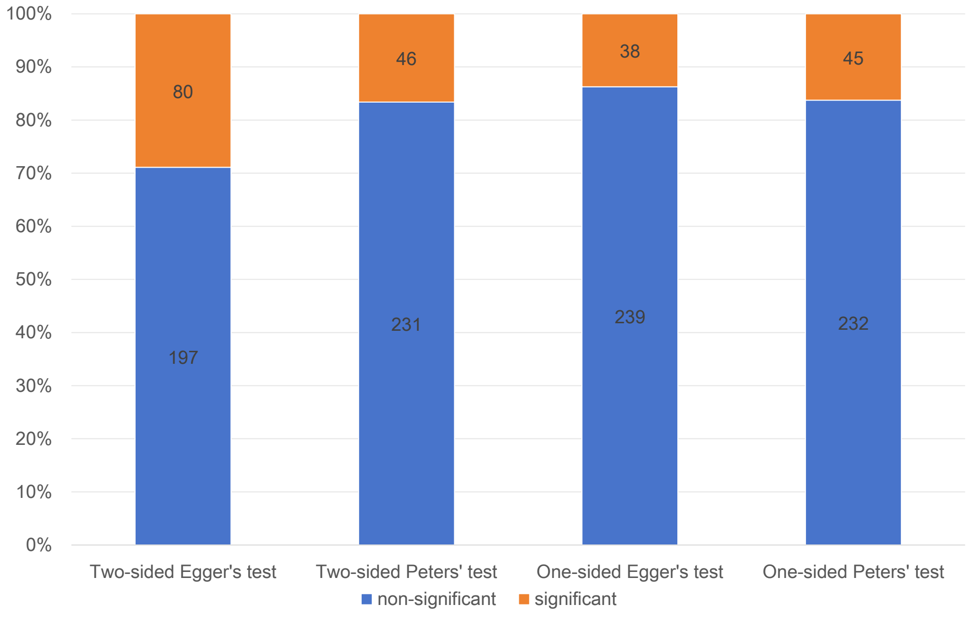

Fig. S1. Proportion of significant or non-significant results in different groups.

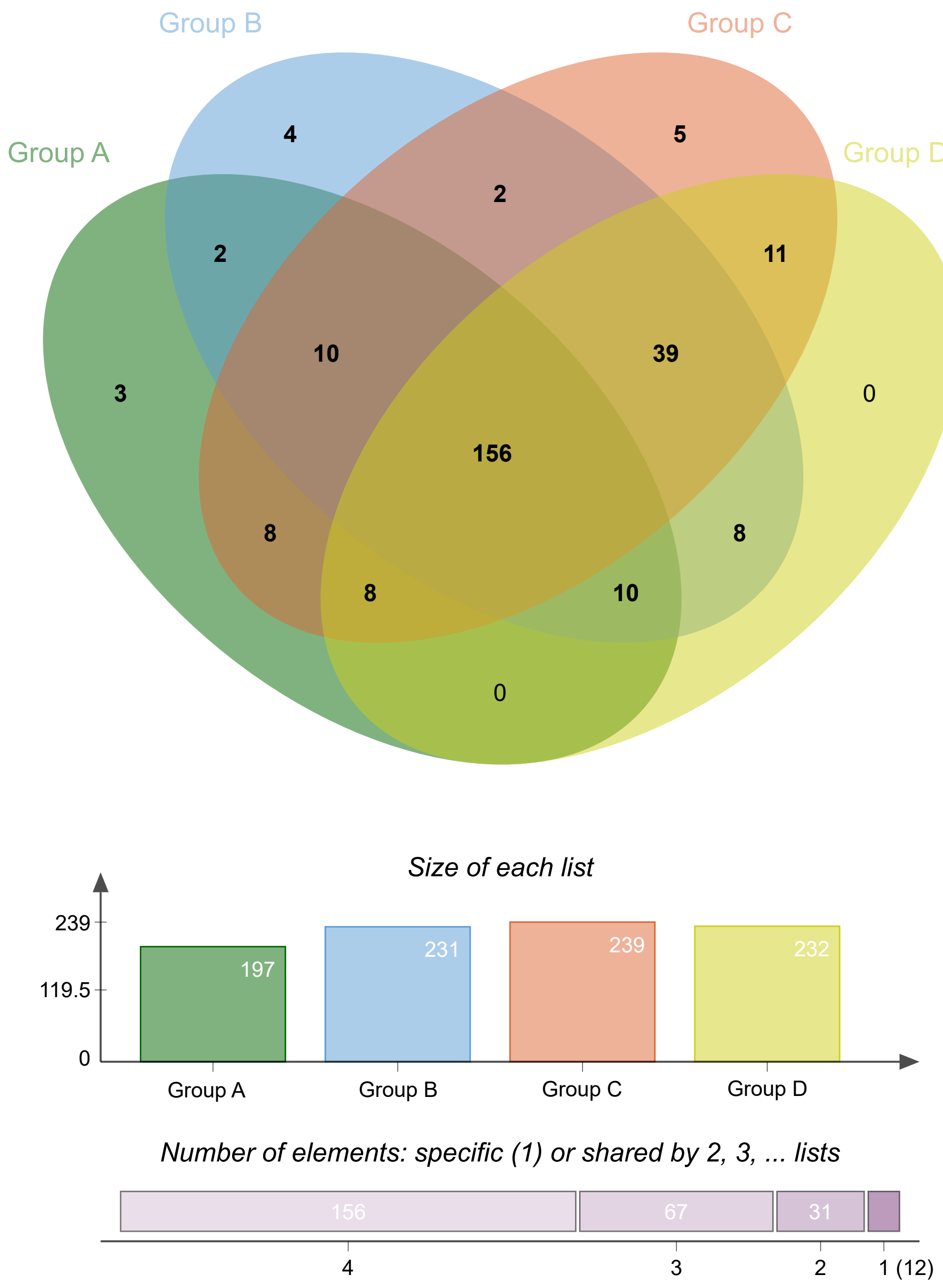

Fig. S2. Venn diagram depicting four types of quantitative assessments of non-significant results. Group A: two-sided Egger's test results; Group B: two-sided Peters' test results; Group C: one-sided Egger's test results; Group D: one-sided Peters' test results.

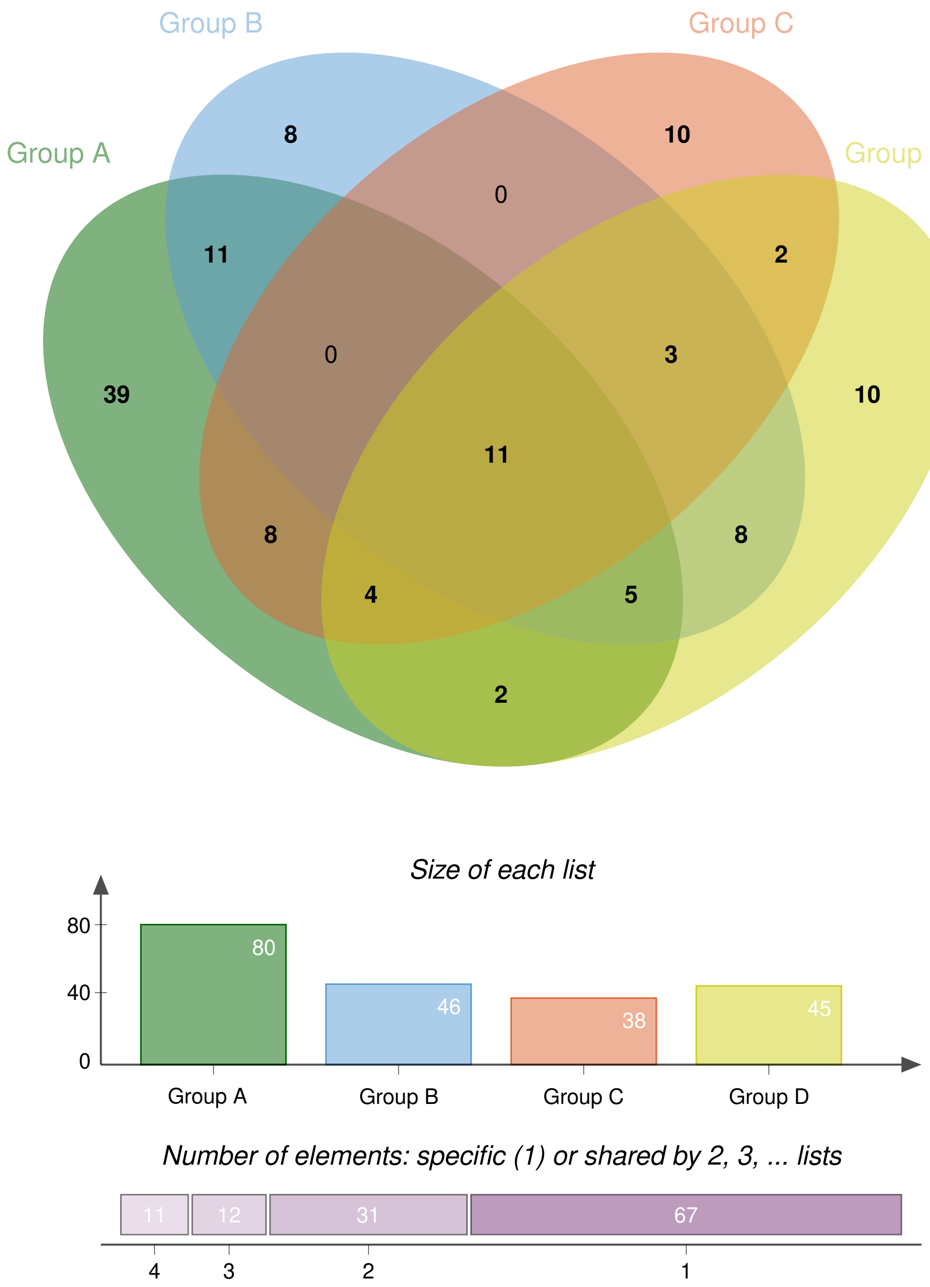

Fig. S3. Venn diagram depicting four types of quantitative assessments of significant results. Group A: two-sided Egger's test results; Group B: two-sided Peters' test results; Group C: one-sided Egger's test results; Group D: one-sided Peters' test results.

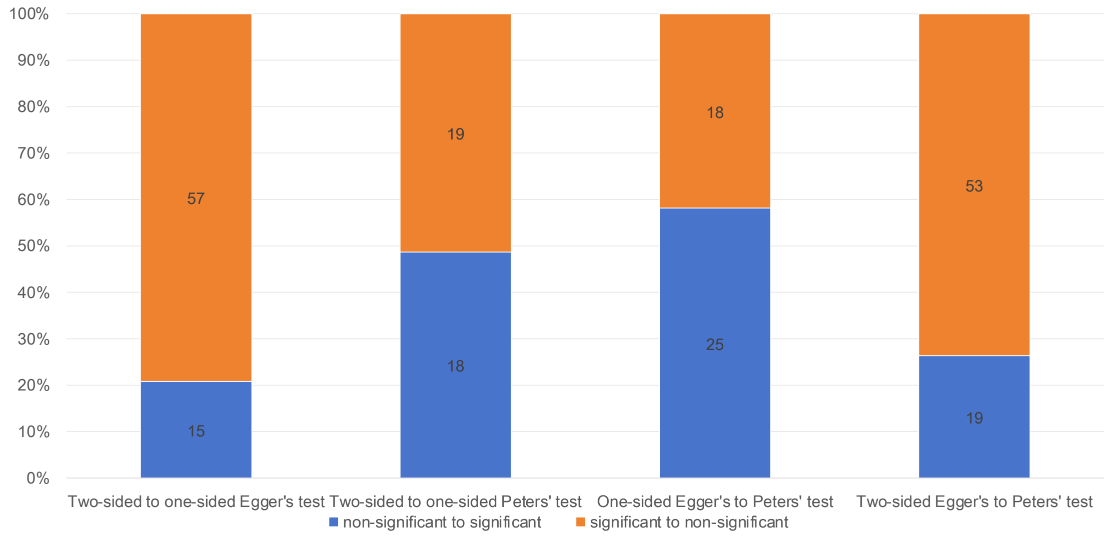

Fig. S4. Proportion of changes in significance across different groups.
